# PFAPA flares observed during COVID outbreak: can emotional stress trigger PFAPA attacks? A multicenter cohort study

**DOI:** 10.1186/s12969-022-00705-7

**Published:** 2022-07-08

**Authors:** Yoel Levinsky, Yonatan Butbul Aviel, Sabreen Abu Ahmad, Mor Broide, Yulia Gendler, Neta Dagan, Michal Gafner, Hadar Gavra, Shelly Kagan, Kfir Kedar, Hamada Mohammad Natour, Rotem Tal, Tamar Veres, Gil Amarilyo, Liora Harel

**Affiliations:** 1 Department of Pediatrics B, Schneider Children’s Medical Center of Israel, Pediatric Rheumatology Unit, Petach Tikva, Israel; 2 Sackler Faculty of Medicine, Tel Aviv University, Tel Aviv, Israel; 3 Pediatric Rheumatology Service, Ruth Rappaport Children’s Hospital, Rambam Health Care Campus, Haifa, Israel; 4 The Ruth and Bruce Rappaport Faculty of Medicine, Technion - Israel Institute of Technology, Haifa, Israel; 5 Department of Pediatrics B, Ruth Rappaport Children’s Hospital, Rambam Medical Center, Haifa, Israel; 6 Department of Pediatrics A, Schneider Children’s Medical Center, Petah Tikva, Israel; 7 Department of Nursing, Ariel University, Ariel, Israel; 8 Pediatric Rheumatology Unit, Schneider Children’s Medical Center of Israel, Petach Tikva, Israel

**Keywords:** PFAPA, Paediatric rheumatology, Emotional stress, Flares

## Abstract

**Objective:**

It is common knowledge among clinicians who treat PFAPA (Periodic Fever, Aphthous Stomatitis, Pharyngitis, Adenitis) patients that emotional stress can trigger PFAPA attacks similarly to other autoinflammatory diseases. However, it has never been proved scientifically. Our aim was to examine whether emotional stress serves as a trigger for PFAPA attacks.

**Methods:**

Patients aged 3-12 years, with active PFAPA, from two Israeli medical centers were enrolled to this study. Patient's parents were reached via phone calls in two occasions: a stressful period related to the COVID-19 pandemic restrictions and a less stressful period. In both times they were asked to report occurrence of PFAPA attacks in the preceding 2 weeks. The relative stress levels of the two periods were validated by an emotional distress scale questionnaire. The significance level was set at 0.05.

**Results:**

Mean age was 7.28 ± 2.7 for the 99 paediatric patients enrolled in the study. Scores for the mean emotional distress questionnaire were statistically significant higher in the stressful period compared to the less stressful period (35.6 ± 8.1 vs. 32.1 ±7.7, respectively, *P* = 0.047). In the stressful period, 41 (38.7%) reported at least one attack during the preceding 2 weeks, compared to 24 (22.6%) in the less stressful period (p = 0.017).

**Conclusion:**

PFAPA flares during COVID-19 outbreak are described. This study is the first to suggest that emotional stress is associated with PFAPA attacks.

**Supplementary Information:**

The online version contains supplementary material available at 10.1186/s12969-022-00705-7.

## Introduction

Periodic fever, aphthous stomatitis, pharyngitis and cervical adenitis (PFAPA) is one of the most common autoinflammatory syndrome. It was reported in 1987 by Marshall et al., who described recurrent episodes of fever along with pharyngitis, cervical lymph nodes, and mouth ulcers mimicking cyclic neutropenia, but with a normal neutrophil count [1]. The exact prevalence of this syndrome is unknown, but PFAPA appears to be more common than other periodic fever diseases [2].

A typical PFAPA attack usually lasts 3-7 days, with intervals of 2-8 weeks [3]. At certain stages of the disease episodes may occur at regular intervals, with predictable attacks [4]. Medications may reduce the frequency of attacks, especially glucocorticoids therapy where a single dose usually halts the attack within hours, but in about half of patients frequency of attacks increases [5]. Our group recently found that a Mediterranean ancestry may play a role in the development of the disease [6]; however, other factors that affect the development or severity of the disease remain unknown.

We and others have clinically observed that emotional stress may play a significant role in the development of PFAPA attacks. However, periodic emotional stress as a contributor to an immunologic burst is hard to prove using scientific tools, especially in toddlers, although the association between psychological factors and attacks in patients with familial Mediterranean fever (FMF) is well-established [7].

In March 2020, during COVID-19 pandemic outbreak, a general lockdown was announced in Israel, closing all educational institutions and work places for approximately 2 months. Subsequently and on short notice (~2 weeks), preschools and schools were reopened. We assumed that familial and personal stress due to COVID-19 outbreak consequences, along with returning to school (considered a significant stressor in children [8]) on short notice may have served as a stressful event to the patients, and therefore as a trigger for induction of flare.

The objectives of this study were to examine whether emotional stress triggers PFAPA attacks. A secondary goal was to examine whether parents of children with PFAPA identify specific environmental triggers for their children's flares.

## Methods

### Participating patients and proof of concept

The study was conducted among children with PFAPA who attend the paediatric rheumatology division of two, large, tertiary-level medical centers in Israel (Schneider Children's Medical Center of Israel, Ruth Rappaport’s Children's Hospital). Inclusion criteria were all patients aged 3-12 years, diagnosed with PFAPA by a paediatric rheumatologist during January 2016 - February 2020. The diagnosis was based on clinical features consist with PFAPA (including fever as a mandatory feature), clinical response to glucocorticoids, and absence of other autoinflammatory manifestations. Exclusion criteria included children with a diagnosis of another periodic fever disease, such as FMF or other rheumatic disease, and children treated with steroids for other reasons. In addition, patients who did not experience flares during the 6 months prior to study enrollment were excluded.

This study included 2 steps: (1) identification of a potential "stressful period", and "less-stressful period" to be used as a control; (2) documentation of PFAPA flares, and questioning parents on identification of environmental triggers for their children's flares.

In order to standardize comparisons, we required the stressful and less stressful periods be generalized to the study population (i.e., at national scale), at the same time point. The stressful period chosen was the week of May 21-27, 2020, corresponding to the end of the "first wave" of COVID-19 in Israel, and starting a few days after the resumption of studies in kindergartens and schools on May 17, 2020, after a short notice prior to returning to school. We assumed these events constituted a period of stress for the children. The less stressful period chosen was August 17-24 week in the middle of summer break, when the COVID-19 outbreak was at a nadir and the economy almost returned to normal [9]. We assumed this period was relatively less stressful. Relative stress levels in the above mentioned periods were validated by participating parents filling out an emotional distress questionnaire (discussed below).

In order to avoid recall bias, interviewers were instructed not to provide the specific aims of the study.

### Protocol

Patients were contacted via phone calls during the stressful and less stressful periods. A flow chart of the survey is presented in Supplementary Figure S1. After receiving informed consent, parents were asked if their children had experienced PFAPA attacks in the past 6 months, in order to exclude patients on remission for more than 6 months. Eligible parents were then asked if their children had experienced PFAPA attacks in the prior two weeks, and then invited to complete the study questionnaire using Research Electronic Data Capture (REDCap) software, hosted at Clalit Health Services, the largest health maintenance organization (HMO) in Israel. Parents were provided a unique passcode, and a link to the questionnaire was sent to the email address provided in the phone conversation.

The study questionnaire included questions related to the basic clinical aspects of the child's disease, followed by an open question regarding possible triggers of child's attacks, and then the paediatric emotional distress scale (PEDS) [10], a validated questionnaire that has been used for children in various studies [11–13]. This is a tool designed to rely on assessments from parents, who are asked to report prevalence of 21 behavioral problems. Each item is scored on a scale of 1-4 points, ranging from 1 ('almost never') to 4 ('very often'). The scale includes 17 general behavior and four trauma-specific items.

In the second phone call (during the less stressful period), parents were asked if their children had experienced PFAPA attacks in the prior two weeks and then were asked to re-fill the study questionnaires.

The study was approved by the Research Ethics Board of Rabin Medical Center (approval no. RMC-20-0354).

### Statistical analysis

Data were analysed descriptively; according to the distribution appropriate measures of central tendency and dispersion were used. Differences between groups were analysed using paired t-test, or chi-square test, according to the measurement level and underlying distribution. The analysis was made among only those who participated in the two phone-calls. Mean PEDS score of the whole population was compared between the two periods. The significance level was set at 0.05. All analyses were performed using IBM SPSS Statistics for Windows, Version 25.0. Armonk, NY: IBM Corp.

## Results

**Fig. 1:**
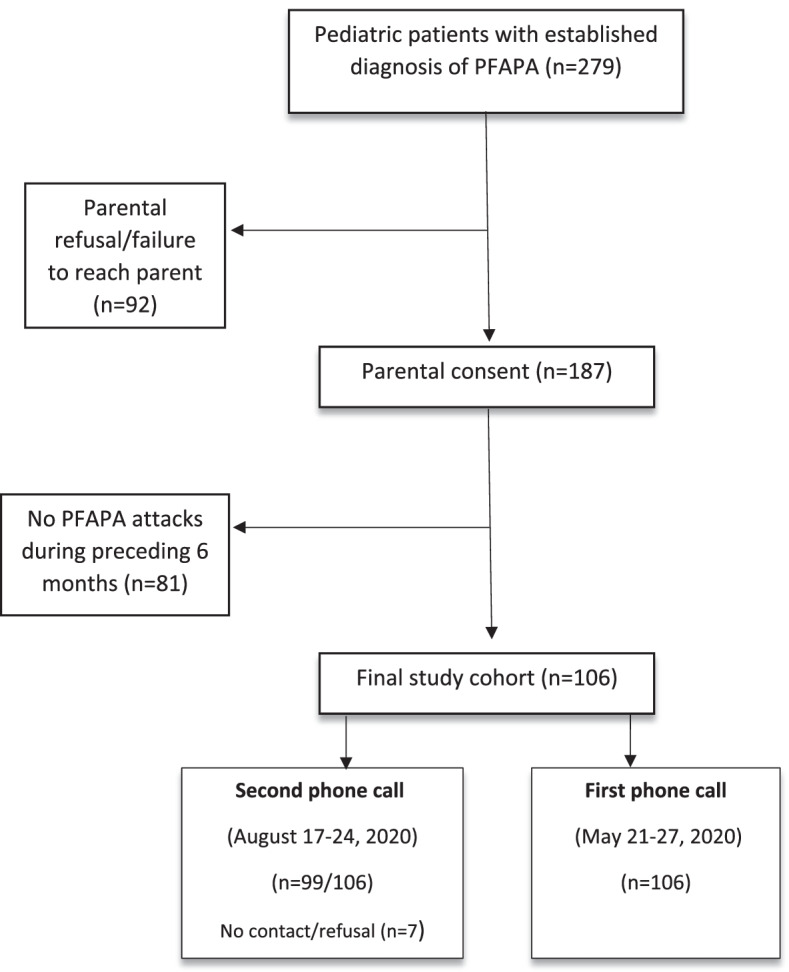
Enrolment flow chart

Of 279 potentially eligible patients, 187 were reached and 81 excluded due to their child being in remission for at least 6 months prior; 106 patients' parents were enrolled and filled the first questionnaire; and 99 parents filled out the second questionnaire. The enrollment flow chart is depicted in Fig. 1.

Table 1 summarizes the sociodemographic and clinical information regarding the paediatric patients in the study: 58/99 (58.5%) were male, average age was 7.28 ± 2.7 years, and 60 (60.6%) attended the early years of elementary school at time of enrollment. Most (91, 91.9%) paediatric patients were treated with single dose of a corticosteroid during attacks, and the mean duration of attacks when treated with corticosteroids was 5.2 ±11.5 hours.

**Table 1:** Sociodemographic and clinical characteristics of children with PFAPA who participated in the study

Characteristic	Value
Number (n)	99
Female sex, n (%)	41 (41.4)
Age at first call (y), mean±SD	7.28± 2.7
Education level	
Kindergarten, n (%)	26 (26.2)
Elementary school (grades 1-3), n (%)	60 (60.6)
≥4^th^ grade, n (%)	13 (13.1)
Symptoms of attacks^a^	
Sore throat, n (%)	74 (74.7)
Overt pharyngitis/tonsillitis, n (%)	61 (61.6)
Abdominal pain, n (%)	60 (60.6)
Legs pain, n (%)	56 (56.6)
Cervical lymphadenopathy, n (%)	51 (51.5)
Oral aphthae, n (%)	39 (39.4)
Steroids treatment during attacks, n (%)	91 (91.9)
Preventive treatment (colchicine), n (%)	9 (9.1)
Attack duration, with treatment (hours), mean±SD	5.2±11.5

^a^According to parents' reports in the questionnaire

On the paediatric emotional distress scale, during the stressful period the mean paediatric emotional distress scale score was statistically significant higher than in the subsequent less stressful period (35.6 ± 8.1 vs. 32.1 ± 7.7, respectively, P <0.047), as shown in Table 2. This served to validate that the timing of the first and second phone calls corresponded with our assumption of stressful and less stressful periods. The distribution of paediatric emotional distress scale among the patients in the 2 different periods is illustrated in supplementary figure S2.

**Table 2:** Attacks status during prior 2 weeks during stressful or less stressful event

Characteristic	First call (May 2020)	Second call (August 2020)	*p*-value
Paediatric emotional distress score (PEDS)^9^, mean (±SD)	35.6 (±8.1)	32.1 (±7.7)	0.047*^a^
Any attacks during last 2 week, n (%)	41 (39.8)	24 (24.2)	0.017*^b^
Number of attacks			0.04*^b^
One, n (%)	27 (25.5)	19 (17.9)	
Two, n (%)	14 (13.2)	5 (4.7)	

* Statistically significant results

^a^Paired-Samples t-test; ^b^Χ^2^ test

More PFAPA attacks were recorded during the stressful compared to the less stressful period, with 41 (38.7%) of parents reporting at least one PFAPA attack in the preceding two weeks to the first call, compared to only 24 (22.6%) prior to the second call (*p* = 0.017). Moreover, a statistically significant number of patients reported more than one attack during the stressful period, as compared to the less stressful period (14, 13.2% vs. 5, 4.7%, respectively, *P* = 0.04).

Finally, the majority 62 (58.5%) of parents reported triggers for PFAPA attacks, 30 (28.3%) reported no triggers, and 14 did not answer the question. Among parents who reported triggers, the most common (45, 72.6%) were emotional triggers, with 14 parents identifying other triggers: physical activity -5, seasonal - 4, changes in temperature - 2, contact with water - 1, type of food - 1, and exposure to the sun - 1.

### Missing data

Among the 279 first phone calls, 92 parents refuse to participate or were failure to reach. Their child's average age was 8.1 ± 3, and Most of them were boys (48, 52.2%). In comparison to the 106 children who participated in the first call, these differences were not statistically significant. Since they did not participate, it is unknown how many of them had an active disease in the time of the call.

## Discussion

After achieving validation of, and comparing stressful and less stressful periods, we were able to show that a higher percentage of children experienced at least one PFAPA attack during the first stressful period, as compared to the second less stressful period (control). Furthermore, the number of attacks was significantly higher during the stressful period. In addition, the majority of parents reported emotional factors as triggers for attacks.

Although development of PFAPA attack in response to emotional stress is a frequent complaint from the parents in our clinic (e.g. 1^st^ day of school, birthday celebration, familial vacation, anxiety due to fight with a friend or a parent), it was never evaluated. The COVID-19 outbreak served as an opportunity to assess this clinical observation due to widespread emotional and economic crisis at the national level, therefore standardizing the events and their timing, and thus minimizing potential bias.

We believe these findings demonstrate, with a high degree of probability, that mental factors such as stress or excitement are triggers for PFAPA attacks.

One of the strengths of the work is the use of relatively objective tools to test the hypothesis. Instead of relying only on a questionnaire based on retrospective self-reporting, which may be biased by previous beliefs or different perceptions of the disease by parents, we chose to contact the parents at a time which we assumed was mentally stressful for the children, and initially asked a simple and objective question ("Have there been attacks in the last two weeks"). Only later, to reinforce the findings, we presented the open question regarding possible triggers and submitted the PEDS questionnaire.

Recent studies have shown that the proportion of children presenting with psychological problems during the COVID-19 pandemic was high and it is likely that the pandemic is worsening the mental health of youth [14]. Leeb et al. reported that the proportion of pediatric ED visits due to mental health issues during the "first wave" of the COVID-19 pandemic increased by 24% for children 5-11 years compared to the same periods in the past [15]. This period is parallel with our study period. During the pandemic there was an increase in hospital mental health related admissions among children and adolescents [16, 17].

The information on triggers for PFAPA attacks is scarce. Although PFAPA attacks tend to occur on a regular cycle, in the first report on the syndrome Marshal et al. commented that intervals between attacks may vary between and among patients, although they found no seasonal variations or association with infections [1]. Many parents report on irregular frequency, for example, to intermittently skip an episode of fever, [5] and the treatment with glucocorticoids may also varies the intervals between attacks [5]. Mean age of patients in our study is higher than typical PFAPA age, which may explain majority of the patients had the disease for a while and it is possible that in later years in the course of the disease regular periodicity is not seen. Environmental factors such as lack of breastfeeding or maternal smoking were found to be associated with the onset of the disease in a small study, but were not examined as triggers of attacks [18]. Padgett reported menstruation as a trigger for PFAPA attacks in an adolescent girl [19]. In addition, a claim was raised regarding the role of the microbes of the tonsils as a trigger for attacks [20], but the issue has not yet been unequivocally proven [21].

The fact that mental state is a trigger for an attack of periodic fever is known in other diseases, especially FMF. In that disease, the frequency of attacks is less predictable than for PFAPA^4^, and several attempts have been made to identify triggers for flares. Karadag et al. conducted a questionnaire study that showed 49.8% of patients (mostly adults) identified psychological stress as a trigger for attacks [22]. Yenokyan et al. found a positive association between FMF attacks and stress events in the two days preceding them [23]. For children with FMF, it was found that psychosocial conditions may explain 27% of the variability of attacks [24]. Another study found that the mean depression and anxiety scores of FMF paediatric patients were positively correlated with the number of attacks [25].

The biological explanation for the effect of stress on FMF attacks may be related to activation of the sympathetic system, initiating the release of catecholamines (epinephrine and norepinephrine), which leads to the secretion of Il-1β, activation of the inflammasome via activation of cAMP signaling pathway protein kinase A (PKA), and an increase in gene expression of REDD1 (regulated in development and DNA damage responses) [7]. The pathogenesis of PFAPA involves the activation of IL-1β as well as inflammasome [26], and therefore it is possible that the effect of emotional triggers is similar to what was found with FMF.

### Limitations

This study has a number of limitations. First, it can be argued that the correlation found between the stressful event and the prevalence of attacks is not indicative of a causal relationship, but may be related to other factors that were not tested (such as the presence of viral infections or the family schedule during holidays). It could also be claimed that the presence of PFAPA attacks influenced the emotional distress scale (and not the opposite). Exposure to common infections may, theoretically, trigger auto-inflammatory attacks or be perceived by the parents as such. Ideally, the comparison may be between the same periods of the year. Nevertheless, in this unique scenario of the COVID-19 pandemic we believe that this confounder is less significant, as it has been shown that the spread of infections (especially respiratory) declined during the first lockdown due to isolation measures and social distancing related to the pandemic [27]. This is difficult to prove, though, in our specific population.

In addition, it is possible that parents' reporting of their personal impressions of triggers for attacks is not objective, and may be biased by cultural, mental and other perceptions. Worth noting, these findings were presented only as reinforcement to the primary outcome. Another problem is defining attacks as PFAPA, which relied on parental reporting. However, the paediatric patients presented with a clinical picture matching PFAPA and their attacks were responsive to glucocorticoids. It can also be claimed that colchicine treatment affects the frequency of attacks, but only 10.4% of our patients were treated with this drug; In addition we performed a sensitivity analysis omitting these patients, and the results were still significant.

In the current study we did not ask about the "regularity" of attacks or its predictable nature, which could be relevant to the susceptibility of the specific child to potential triggers. Future studies are needed to address this issue. Finally, it is important to note that the difference between the PEDS score in the 2 periods was close to the significance level, although still significant.

## Conclusion

This study reports PFAPA flares observed during COVID outbreak in Israel. These results show that emotional distress is associated with higher frequency of PFAPA attacks, thus may be considers as environmental trigger for PFAPA attack. Future research is needed to confirm these findings and characterize additional triggers.

## Supplementary Information

**Additional file 1: Supplementary Figure S1.** The study questionnaire.

**Additional file 2: Supplementary Figure S2**. The distribution of paediatric emotional distress scale (PEDS) scores among the cohort in the 2 different periods. X axis represent patients' serial numbers, Y axis represents PEDS scores.

## Data Availability

The datasets used and/or analysed during the current study are available from the corresponding author on reasonable request.

## Abbreviations

PFAPA: Periodic fever, aphthous stomatitis, pharyngitis and cervical adenitis
FMF: Familial Mediterranean Fever
PEDS: The paediatric emotional distress scale
