# Supplementary figures and images for: PFAPA flares observed during COVID outbreak: can emotional stress trigger PFAPA attacks? A multicenter cohort study
Source: Pediatr Rheumatol Online J. 2022 Jul 8;20:46. doi: 10.1186/s12969-022-00705-7 (PMC9264301; doi:10.1186/s12969-022-00705-7)

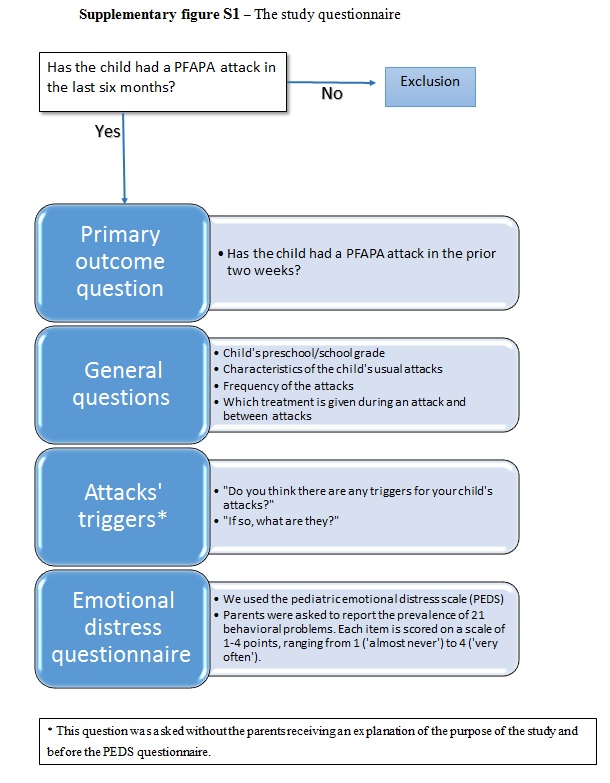

Supplement: Supplementary file 1 — Additional file 1: Supplementary Figure S1. The study questionnaire. [file 12969_2022_705_MOESM1_ESM.jpg]

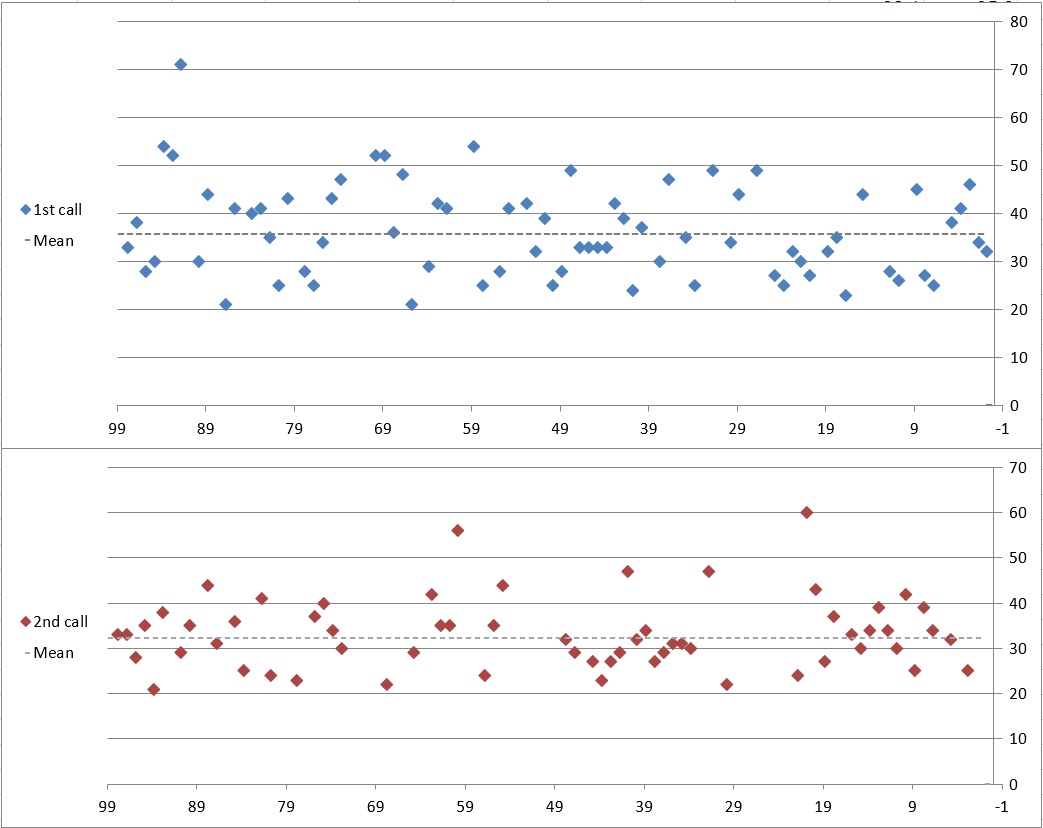

Supplement: Supplementary file 2 — Additional file 2: Supplementary Figure S2. The distribution of paediatric emotional distress scale (PEDS) scores among the cohort in the 2 different periods. X axis represent patients' serial numbers, Y axis represents PEDS scores. [file 12969_2022_705_MOESM2_ESM.jpg]
